# Synthesis, crystal structure and Hirshfeld surface analysis of a zinc(II) coordination polymer of 5-phenyl-1,3,4-oxa­diazole-2-thiol­ate

**DOI:** 10.1107/S2056989022006922

**Published:** 2022-07-14

**Authors:** Mehribon Pirimova, Batirbay Torambetov, Shakhnoza Kadirova, Abdukhakim Ziyaev, Rajesh G Gonnade, Jamshid Ashurov

**Affiliations:** a National University of Uzbekistan named after Mirzo Ulugbek, 4 University St, Tashkent, 100174, Uzbekistan; b S. Yu. Yunusov Institute of the Chemistry of Plant Substances, Academy of Sciences of the Republic of Uzbekistan, Mirzo Ulugbek Str. 77, 100170, Tashkent, Uzbekistan; c Physical and Materials Chemistry Division, CSIR-National Chemical Laboratory, Pune-411008, India; d Institute of Bioorganic Chemistry, Academy of Sciences of Uzbekistan, M. Ulugbek Str, 83, Tashkent, 100125, Uzbekistan; Universidad de Los Andes, Venezuela

**Keywords:** crystal structure, zinc complex, 5-phenyl-1,3,4-oxa­diazole-2-thiol, coordination polymer, Hirshfeld surface analysis

## Abstract

A new zinc coordination polymer with 5-phenyl-1,3,4-oxa­diazole-2-thiol­ate, namely, *catena*-poly[zinc(II)-bis­(μ_2_-5-phenyl-1,3,4-oxa­diazole-2-thiol­ato)-κ^2^ *N* ^3^:*S*;κ^2^ *S*:*N* ^3^], [Zn(C_8_H_5_N_2_OS)_2_]_*n*_, was synthesized. The single-crystal X-ray diffraction analysis shows that the polymeric structure crystallizes in the centrosymmetric monoclinic *C*2/*c* space group. The Zn^II^ atom is coordinated to two S and two N atoms from four crystallographically independent (*L*) ligands, forming zigzag chains along the [001] direction. This polymer complex forms an eight-membered [Zn–S–C–N–Zn–S–C–N] chair-like ring with two Zn^II^ atoms and two ligand mol­ecules. On the Hirshfeld surface, the largest contributions come from the short contacts such as van der Waals forces, including H⋯H, C⋯H and S⋯H. Inter­actions including N⋯H, O⋯H and C⋯C contacts were also observed; however, their contribution to the overall stability of the crystal lattice is minor.

## Abstract 2

The mol­ecular and crystal structure of a zinc coordination polymer with 5-phenyl-1,3,4-oxa­diazole-2-thiol­ate were studied and Hirshfeld surfaces and fingerprint plots were generated to investigate various inter­molecular inter­actions.

## 1. Chemical context

Among heterocyclic organic compounds, 1,3,4-oxa­diazo­les have become an important class of heterocycles because of their broad spectrum of biological activity (De Oliveira *et al.*, 2012 ▸; Vaidya *et al.*, 2020 ▸). Scientists have identified many properties of 1,3,4-oxa­diazole derivatives, such as anti­microbial (Bala *et al.*, 2014 ▸; Zachariah *et al.*, 2015 ▸; Ahmed *et al.*, 2017 ▸; Razzoqova *et al.*, 2019 ▸ **)**, anti­tuberculosis (Makane *et al.*, 2019 ▸; Wang *et al.*, 2022 ▸), anti­cancer (Alam, 2022 ▸; Vaidya *et al.*, 2020 ▸; Zhang *et al.*, 2005 ▸), anti-inflammatory (Abd-Ellah *et al.*, 2017 ▸), analgesic (Husain & Ajmal, 2009 ▸), herbicidal (Sun *et al.*, 2014 ▸; Duan *et al.*, 2011 ▸) and anti­fungal (Zhang *et al.*, 2013 ▸; Capoci *et al.*, 2019 ▸) activities. Heterocyclic thio­nes are an important type of compound in coordination chemistry because of their potential multifunctional donor sites, namely either exocyclic sulfur or endocyclic nitro­gen (Reddy *et al.*, 2011 ▸; Wang *et al.*, 2010 ▸). The presence of the 1,3,4-oxa­diazole ring affects the physicochemical and pharmacokinetic properties of the entire compound. An exciting feature of these metal complexes is that they can be mononuclear (Singh *et al.*, 2008 ▸; Ouilia *et al.*, 2012 ▸), binuclear (Xiao *et al.*, 2011 ▸; Wang *et al.*, 2007 ▸) and/or polymeric (Beghidja *et al.*, 2007 ▸).

Oxa­diazole ligands are ideal objects for creating new coordination compounds with great potential in various fields. Scientists have written extensive literature on the biological properties of oxa­diazole-based complex compounds, especially on their anti­cancer effects. In addition to these, in the field of electrical engineering, metal complexes bearing oxa­diazole ligands have been used as emitting particles in light-emitting diodes. The introduction of various functionalized oxa­diazole ligands makes it easy to control the emission color, thermal stability, and film-forming properties of such complexes (Salassa & Terenzi, 2019 ▸). 
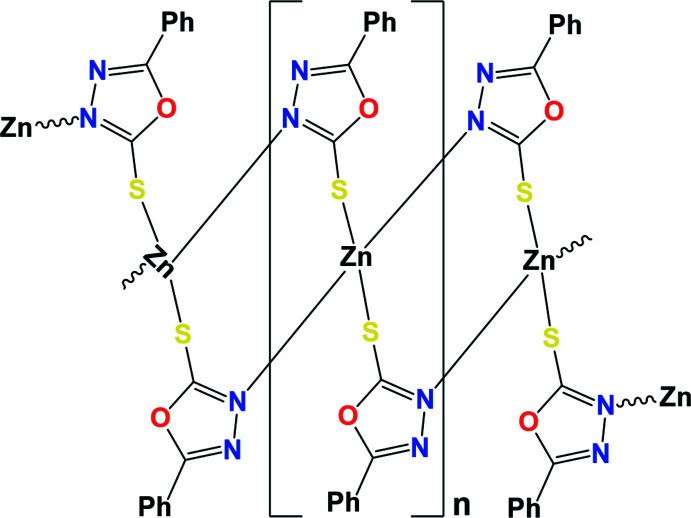


Herein, we report on the synthesis and crystal structure of a new polymeric complex, [Zn*L*_2_]_*n*_, with *L* = 5-phenyl-1,3,4-oxa­diazole-2-thiol.

## 2. Structural commentary

**Figure 1:**
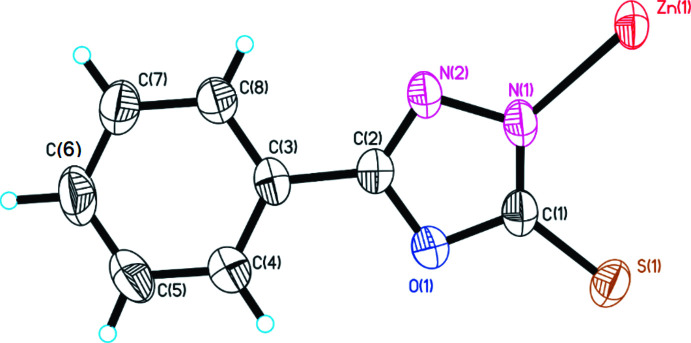
The mol­ecular structure of [Zn_0.5_ *L*] with the atom-numbering scheme. Displacement ellipsoids are drawn at the 50% probability level and H atoms are displayed as small spheres of arbitrary radii.

**Figure 2:**
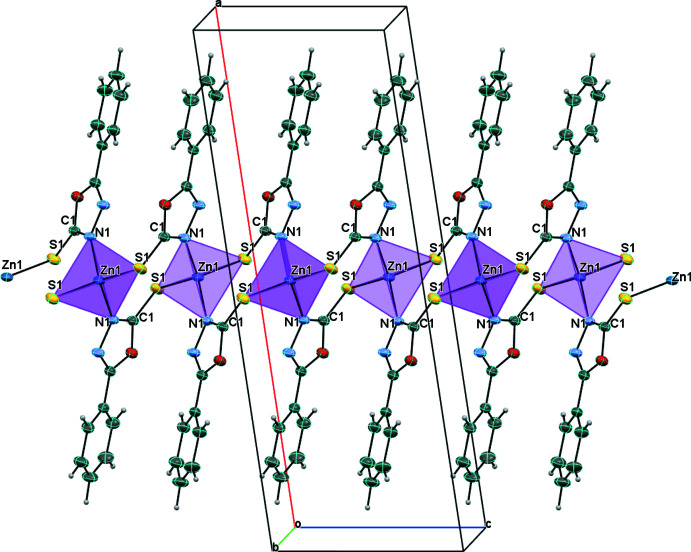
The view of the mol­ecular packing showing the polymeric chain extended along the *c*-axis.

The single crystal X-ray structure of 5-phenyl-1,3,4-oxa­diazole-2-thiol­ate Zn^II^ shows a polymeric structure that crystallizes in the centrosymmetric monoclinic space group *C*2/*c* (Table 2 ▸). As seen in Fig. 1 ▸, its asymmetric unit contains half a zinc atom and one ligand anion. The central Zn^II^ atom has a distorted tetra­hedral environment comprising two sulfur and two nitro­gen atoms. It is coordinated by four crystallographically independent (*L*) ligands, forming zigzag chains along the [001] direction, which are linked by two sulfur atoms and two nitro­gen atoms of four ligands. The Zn1—S1 and Zn1—N1 bond lengths are 2.3370 (5) Å, 2.0184 (14) Å, respectively. In this case, the bond angles of the atom forming the tetra­hedral polyhedron are slightly different from the angles of the ideal tetra­hedron [N1—Zn1–N1 = 111.37 (9)°, S1—Zn1—S1 = 100.46 (3)° and N1—Zn1—S1 = 108.51 (4)°]. It is known from the literature (Razzoqova *et al.*, 2019 ▸) that the sulfur atom in the 1,3,4-oxadiazole-2-thione mol­ecule is attached to the ring by a double bond. In this polymer complex synthesized based on Zn^II^ ion, the oxa­diazole derivative transforms into the thiol tautomeric form and binds to the Zn ion. The N1 atom in the ligand mol­ecule, on the other hand, forms a bond with another Zn^II^ ion due to its high electron-donating property, resulting in an eight-membered [Zn–S–C–N–Zn–S–C–N] chair-like ring with two Zn^II^ atoms and two ligand mol­ecules (Fig. 2 ▸). The dihedral angle between the mean planes of the phenyl (C3–C8) and oxa­diazole (C1/O1/C2/N2/N1) rings of the ligand mol­ecule is 13.42 (8)°. The conformation of the oxa­diazole-thiol fragment of the ligand is approximately planar (r.m.s. deviation 0.006 Å), with a maximum deviation from the least-squares plane of 0.009 (1) Å for atom O1. The dihedral angle between the planes of the two neighboring independent oxa­diazole-thiol (C1/O1/C2/N2/N1/S1) fragments is 64.10 (9)°.

## 3. Supra­molecular features

**Figure 3:**
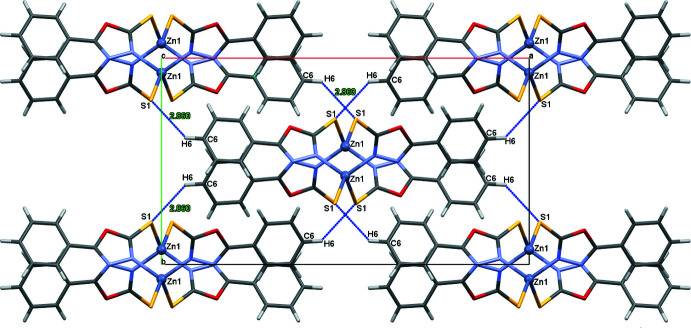
Crystal packing of the polymeric chains in the [(Zn*L*_2_)_*n*_] structure. The projection is along the [001] direction. Hydrogen bonds are shown by cyan lines.

The [(Zn*L*_2_)_*n*_] unit is given as a monomer of the polymeric chain that extends parallel to the *c*-axis. Along the polymeric chain, the hydro­philic groups are concentrated within the core of the chain while the phenyl rings project approximately normal to the chain. Neighboring chains across the *ab* plane are loosely connected *via* a rather weak C6—H6⋯S1 hydrogen bond (Table 1 ▸, Fig. 3 ▸).

## 4. Hirshfeld surface analysis

**Figure 4:**
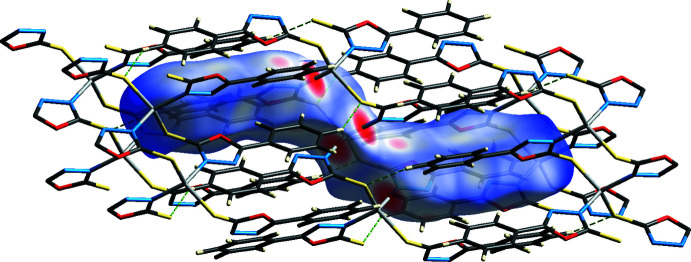
Hirshfeld surfaces mapped over *d*_norm_ calculated for the monomer part of the polymer mol­ecule.

To further investigate the inter­molecular inter­actions present in the title compound, a Hirshfeld surface analysis was performed, and the two-dimensional fingerprint plots were generated with *CrystalExplorer17* (Turner *et al.*, 2017 ▸). The Hirshfeld surface mapped over *d*_norm_ and corresponding colors representing various inter­actions are shown in Fig. 4 ▸. We chose the Zn*L*_2_ mol­ecular fragment as the monomer unit for calculating the Hirshfeld surface of this polymer complex.

**Figure 5:**
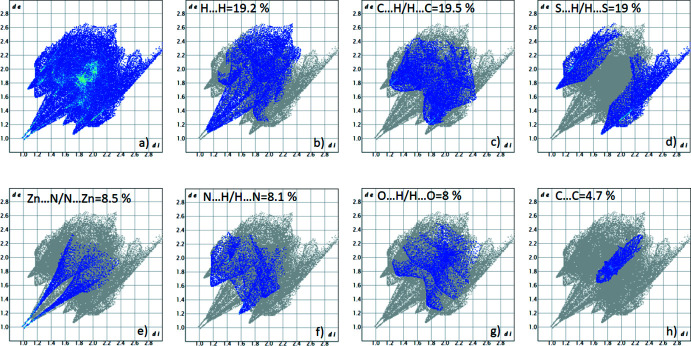
Contributions of the various contacts to the fingerprint plot built using the Hirshfeld surface of the title compound.

The large red areas on the Hirshfeld surface correspond to the Zn⋯N inter­actions. The two-dimensional (2D) fingerprint plots (McKinnon *et al.*, 2007 ▸) are shown in Fig. 5 ▸. On the Hirshfeld surface, the largest contributions (19.2%, 19.5% and 19%) come from short contacts such as van der Waals forces, H⋯H, C⋯H and S⋯H contacts. N⋯H (8.1%), O⋯H (8%) and C⋯C (4.7%) contacts are also observed. These inter­actions play a crucial role in the overall stabilization of the crystal packing.

## 5. Database survey

A survey of the Cambridge Structural Database (CSD, version 5.43, update of November 2021; Groom *et al.*, 2016 ▸) revealed that crystal structures had been reported for complexes of 1,3,4-oxa­diazole derivatives and a number of metal ions, including zinc, copper, nickel, manganese, cadmium, cobalt and silver. No polymer complexes containing [*M*–S–C–N–*M*–S–C–N] eight-membered cyclization have been reported. The structures of complexes of Pt, Sn and Au based on 5-phenyl-1,3,4-oxa­diazole-2-thiole with additional ligands have been deposited in the CSD (FATNIZ, Al-Jibori *et al.*, 2012 ▸; HAXTAC, Ma *et al.*, 2005 ▸; and YIVVEG, Chaves *et al.*, 2014 ▸). However, no complexes containing only the zinc ion and 5-phenyl-1,3,4-oxa­diazole-2-thiol­ate have been documented in the CSD.

## 6. Synthesis and crystallization

ZnCl_2_ (0.136 g, 0.001 mol) and 5-phenyl-1,3,4-oxa­diazole-2-thiol (ligand) (0.354 g, 0.002 mol) were dissolved separately in ethanol (10 mL). To a solution of the ligand, an aqueous solution of KOH (0.112 g, 0.002 mol) was added. The obtained solutions were mixed together and stirred at 323 K for 20 min. A white precipitate was obtained. The precipitate was filtered and allowed to dry. The solid residue was dissolved in DMF to crystallize for the single crystal X-ray diffraction studies. X-ray quality single crystals were produced after 10 days by slow evaporation of the solution.

## 7. Refinement

Crystal data, data collection and structure refinement details are summarized in Table 2 ▸. All the hydrogen atoms were located in difference-Fourier maps and refined isotropically.

## Supplementary Material

Crystal structure: contains datablock(s) I. DOI: 10.1107/S2056989022006922/dj2049sup1.cif

Structure factors: contains datablock(s) I. DOI: 10.1107/S2056989022006922/dj2049Isup2.hkl

CCDC reference: 2184492

Additional supporting information:  crystallographic information; 3D view; checkCIF report

## Figures and Tables

**Table 1:** Hydrogen-bond geometry (Å, °)

*D*—H⋯*A*	*D*—H	H⋯*A*	*D*⋯*A*	*D*—H⋯*A*
C6—H6⋯S1^i^	1.00 (3)	2.86 (3)	3.608 (2)	132 (2)

Symmetry code: (i) 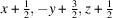 .

**Table 2:** Experimental details

Crystal data	
Chemical formula	[Zn(C_8_H_5_N_2_OS)_2_]
*M*_r_	419.79
Crystal system, space group	Monoclinic, *C*2/*c*
Temperature (K)	293
*a*, *b*, *c* (Å)	20.4223 (3), 11.3260 (2), 7.4019 (1)
β (°)	98.310 (1)
*V* (Å^3^)	1694.11 (5)
*Z*	4
Radiation type	Cu *K*α
μ (mm^−1^)	4.48
Crystal size (mm)	0.60 × 0.14 × 0.08

Data collection	
Diffractometer	XtaLAB Synergy, single source at home/near, HyPix3000
Absorption correction	Multi-scan (*CrysAlis PRO*; Rigaku OD, 2020 ▸)
*T*_min_, *T*_max_	0.099, 1.000
No. of measured, independent and observed [*I* > 2σ(*I*)] reflections	7033, 1634, 1536
*R*_int_	0.028
(sin θ/λ)_max_ (Å^−1^)	0.615

Refinement	
*R*[*F* ^2^ > 2σ(*F* ^2^)], *wR*(*F* ^2^), *S*	0.026, 0.073, 1.09
No. of reflections	1634
No. of parameters	134
H-atom treatment	All H-atom parameters refined
Δρ_max_, Δρ_min_ (e Å^−3^)	0.26, −0.33

Computer programs: *CrysAlis PRO* (Rigaku OD, 2020 ▸), *SHELXT2014/5* (Sheldrick, 2015*a* ▸), *SHELXL2016/6* (Sheldrick, 2015*b* ▸) and *OLEX2* (Dolomanov *et al.*, 2009 ▸).

## supplementary crystallographic information

### Crystal data

**Table:**

[Zn(C_8_H_5_N_2_OS)_2_]	*F*(000) = 848
*M**_r_* = 419.79	*D*_x_ = 1.646 Mg m^−^^3^
Monoclinic, *C*2/*c*	Cu *K*α radiation, λ = 1.54184 Å
*a* = 20.4223 (3) Å	Cell parameters from 5128 reflections
*b* = 11.3260 (2) Å	θ = 4.4–71.1°
*c* = 7.4019 (1) Å	µ = 4.48 mm^−^^1^
β = 98.310 (1)°	*T* = 293 K
*V* = 1694.11 (5) Å^3^	Block, colourless
*Z* = 4	0.60 × 0.14 × 0.08 mm

### Data collection

**Table:**

XtaLAB Synergy, single source at home/near, HyPix3000 diffractometer	1634 independent reflections
Radiation source: micro-focus sealed X-ray tube, PhotonJet (Cu) X-ray Source	1536 reflections with *I* > 2σ(*I*)
Mirror monochromator	*R*_int_ = 0.028
Detector resolution: 10.0000 pixels mm^-1^	θ_max_ = 71.5°, θ_min_ = 4.4°
ω scans	*h* = −25→23
Absorption correction: multi-scan (CrysAlisPro; Rigaku OD, 2020)	*k* = −13→13
*T*_min_ = 0.099, *T*_max_ = 1.000	*l* = −9→8
7033 measured reflections	

### Refinement

**Table:**

Refinement on *F*^2^	Primary atom site location: structure-invariant direct methods
Least-squares matrix: full	Hydrogen site location: difference Fourier map
*R*[*F*^2^ > 2σ(*F*^2^)] = 0.026	All H-atom parameters refined
*wR*(*F*^2^) = 0.073	*w* = 1/[σ^2^(*F*_o_^2^) + (0.0425*P*)^2^ + 0.6399*P*] where *P* = (*F*_o_^2^ + 2*F*_c_^2^)/3
*S* = 1.09	(Δ/σ)_max_ = 0.001
1634 reflections	Δρ_max_ = 0.26 e Å^−^^3^
134 parameters	Δρ_min_ = −0.33 e Å^−^^3^
0 restraints	

### Special details

**Table:**

Geometry. All esds (except the esd in the dihedral angle between two l.s. planes) are estimated using the full covariance matrix. The cell esds are taken into account individually in the estimation of esds in distances, angles and torsion angles; correlations between esds in cell parameters are only used when they are defined by crystal symmetry. An approximate (isotropic) treatment of cell esds is used for estimating esds involving l.s. planes.

### Fractional atomic coordinates and isotropic or equivalent isotropic displacement parameters (Å^2^)

**Table:**

	*x*	*y*	*z*	*U*_iso_*/*U*_eq_	
Zn1	0.500000	0.42861 (3)	0.750000	0.03700 (13)	
S1	0.52858 (2)	0.70338 (4)	0.49120 (6)	0.04489 (15)	
O1	0.64980 (6)	0.66497 (11)	0.66937 (16)	0.0378 (3)	
N1	0.57974 (7)	0.52907 (14)	0.7241 (2)	0.0381 (3)	
N2	0.64167 (7)	0.49941 (14)	0.8226 (2)	0.0402 (3)	
C2	0.68103 (8)	0.58151 (15)	0.7854 (2)	0.0360 (4)	
C3	0.75171 (8)	0.59280 (16)	0.8505 (2)	0.0370 (4)	
C1	0.58617 (8)	0.62555 (15)	0.6334 (2)	0.0362 (4)	
C8	0.78515 (9)	0.49670 (18)	0.9351 (3)	0.0442 (4)	
C4	0.78562 (10)	0.69688 (18)	0.8287 (3)	0.0472 (4)	
C7	0.85194 (10)	0.5044 (2)	0.9992 (3)	0.0572 (5)	
C5	0.85239 (11)	0.7043 (2)	0.8940 (3)	0.0578 (5)	
C6	0.88524 (10)	0.6091 (2)	0.9798 (3)	0.0613 (6)	
H8	0.7634 (11)	0.4299 (18)	0.946 (3)	0.042 (6)*	
H5	0.8723 (13)	0.772 (3)	0.874 (3)	0.068 (7)*	
H4	0.7642 (12)	0.761 (2)	0.771 (3)	0.059 (7)*	
H7	0.8758 (14)	0.443 (2)	1.056 (4)	0.074 (8)*	
H6	0.9339 (15)	0.614 (3)	1.020 (4)	0.088 (9)*	

### Atomic displacement parameters (Å^2^)

**Table:**

	*U*^11^	*U*^22^	*U*^33^	*U*^12^	*U*^13^	*U*^23^
Zn1	0.02217 (18)	0.0460 (2)	0.0416 (2)	0.000	0.00048 (13)	0.000
S1	0.0363 (3)	0.0440 (3)	0.0508 (3)	0.00848 (17)	−0.00577 (19)	−0.00479 (18)
O1	0.0309 (6)	0.0384 (6)	0.0423 (6)	−0.0034 (5)	−0.0009 (5)	0.0004 (5)
N1	0.0228 (7)	0.0461 (8)	0.0440 (8)	−0.0019 (6)	0.0001 (6)	0.0003 (6)
N2	0.0243 (7)	0.0483 (8)	0.0462 (8)	−0.0030 (6)	−0.0012 (6)	0.0056 (6)
C2	0.0291 (8)	0.0410 (9)	0.0368 (8)	−0.0004 (6)	0.0010 (7)	−0.0010 (6)
C3	0.0276 (8)	0.0468 (9)	0.0359 (8)	−0.0050 (7)	0.0019 (6)	−0.0033 (7)
C1	0.0273 (8)	0.0418 (9)	0.0386 (8)	0.0008 (7)	0.0013 (6)	−0.0070 (7)
C8	0.0357 (9)	0.0498 (11)	0.0459 (9)	−0.0059 (8)	0.0012 (7)	0.0053 (8)
C4	0.0380 (10)	0.0446 (10)	0.0578 (11)	−0.0048 (8)	0.0027 (8)	0.0008 (9)
C7	0.0363 (10)	0.0697 (14)	0.0620 (12)	0.0004 (10)	−0.0044 (9)	0.0124 (11)
C5	0.0393 (11)	0.0591 (13)	0.0741 (14)	−0.0179 (9)	0.0052 (10)	−0.0027 (11)
C6	0.0288 (10)	0.0797 (15)	0.0722 (14)	−0.0108 (10)	−0.0035 (9)	0.0046 (12)

### Geometric parameters (Å, º)

**Table:**

Zn1—S1^i^	2.3370 (5)	C3—C8	1.385 (3)
Zn1—S1^ii^	2.3370 (5)	C3—C4	1.388 (3)
Zn1—N1	2.0184 (14)	C8—C7	1.381 (3)
Zn1—N1^iii^	2.0184 (14)	C8—H8	0.89 (2)
S1—C1	1.7059 (17)	C4—C5	1.382 (3)
O1—C2	1.371 (2)	C4—H4	0.92 (3)
O1—C1	1.3635 (19)	C7—C6	1.384 (3)
N1—N2	1.4062 (18)	C7—H7	0.92 (3)
N1—C1	1.299 (2)	C5—C6	1.376 (4)
N2—C2	1.285 (2)	C5—H5	0.89 (3)
C2—C3	1.460 (2)	C6—H6	1.00 (3)
			
S1^i^—Zn1—S1^ii^	100.46 (3)	O1—C1—S1	120.26 (13)
N1—Zn1—S1^ii^	108.51 (4)	N1—C1—S1	129.90 (13)
N1—Zn1—S1^i^	113.82 (4)	N1—C1—O1	109.83 (14)
N1^iii^—Zn1—S1^i^	108.50 (4)	C3—C8—H8	119.3 (14)
N1^iii^—Zn1—S1^ii^	113.82 (5)	C7—C8—C3	120.24 (19)
N1—Zn1—N1^iii^	111.37 (9)	C7—C8—H8	120.5 (14)
C1—S1—Zn1^i^	102.39 (6)	C3—C4—H4	120.8 (16)
C1—O1—C2	103.92 (13)	C5—C4—C3	119.6 (2)
N2—N1—Zn1	119.57 (11)	C5—C4—H4	119.6 (16)
C1—N1—Zn1	131.80 (12)	C8—C7—C6	119.6 (2)
C1—N1—N2	108.57 (13)	C8—C7—H7	122.8 (17)
C2—N2—N1	105.05 (14)	C6—C7—H7	117.6 (17)
O1—C2—C3	119.65 (15)	C4—C5—H5	116.4 (17)
N2—C2—O1	112.61 (14)	C6—C5—C4	120.3 (2)
N2—C2—C3	127.75 (16)	C6—C5—H5	123.3 (17)
C8—C3—C2	118.67 (16)	C7—C6—H6	119.8 (18)
C8—C3—C4	119.85 (17)	C5—C6—C7	120.33 (19)
C4—C3—C2	121.48 (17)	C5—C6—H6	119.8 (18)
			
Zn1^i^—S1—C1—O1	116.48 (12)	C2—O1—C1—S1	−179.75 (12)
Zn1^i^—S1—C1—N1	−65.24 (17)	C2—O1—C1—N1	1.66 (18)
Zn1—N1—N2—C2	−176.89 (12)	C2—C3—C8—C7	−179.66 (19)
Zn1—N1—C1—S1	−2.7 (3)	C2—C3—C4—C5	179.34 (19)
Zn1—N1—C1—O1	175.68 (11)	C3—C8—C7—C6	0.5 (3)
O1—C2—C3—C8	−166.60 (16)	C3—C4—C5—C6	0.2 (3)
O1—C2—C3—C4	13.2 (3)	C1—O1—C2—N2	−1.25 (19)
N1—N2—C2—O1	0.4 (2)	C1—O1—C2—C3	178.37 (15)
N1—N2—C2—C3	−179.19 (17)	C1—N1—N2—C2	0.68 (19)
N2—N1—C1—S1	−179.91 (13)	C8—C3—C4—C5	−0.9 (3)
N2—N1—C1—O1	−1.49 (19)	C8—C7—C6—C5	−1.2 (4)
N2—C2—C3—C8	13.0 (3)	C4—C3—C8—C7	0.6 (3)
N2—C2—C3—C4	−167.27 (19)	C4—C5—C6—C7	0.9 (4)

Symmetry codes: (i) −*x*+1, −*y*+1, −*z*+1; (ii) *x*, −*y*+1, *z*+1/2; (iii) −*x*+1, *y*, −*z*+3/2.

### Hydrogen-bond geometry (Å, º)

**Table:**

*D*—H···*A*	*D*—H	H···*A*	*D*···*A*	*D*—H···*A*
C6—H6···S1^iv^	1.00 (3)	2.86 (3)	3.608 (2)	132 (2)

Symmetry code: (iv) *x*+1/2, −*y*+3/2, *z*+1/2.
